# Multifunctional croconaine nanoparticles for efficient optoacoustic imaging of deep tumors and photothermal therapy

**DOI:** 10.1515/nanoph-2022-0469

**Published:** 2022-09-26

**Authors:** Nian Liu, Patrick O’Connor, Vipul Gujrati, Pia Anzenhofer, Uwe Klemm, Karin Kleigrewe, Michael Sattler, Oliver Plettenburg, Vasilis Ntziachristos

**Affiliations:** Chair of Biological Imaging, School of Medicine, Technical University of Munich, Munich 81675, Germany; Institute of Biological and Medical Imaging, Helmholtz Zentrum München (GmbH), Neuherberg 85764, Germany; Department of Nuclear Medicine, PET Center, the First Affiliated Hospital, Zhejiang University School of Medicine, Hangzhou 310003, China; Institute of Medicinal Chemistry, Helmholtz Zentrum München (GmbH), Neuherberg 85764, Germany; Institute of Structural Biology, Helmholtz Zentrum München (GmbH), Neuherberg 85764, Germany; Bavarian Center for Biomolecular Mass Spectrometry (BayBioMS), Technical University of Munich, Freising 85354, Germany; Bavarian NMR Center and Center for Integrated Protein Science Munich at Department of Chemistry, Technical University of Munich, Garching 85747, Germany; Center for Biomolecular Drug Research (BMWZ), Institute of Organic Chemistry, Leibniz Universität Hannover, Hannover 30167, Germany

**Keywords:** 880 nm, croconaine, deep tumors, nanoparticles, optoacoustics, photothermal therapy

## Abstract

The proper design of near-infrared light-absorbing agents enables efficient optoacoustic imaging-guided phototherapy. In particular, several croconaine-based organic agents with excellent optical properties have been recently reported for this purpose. However, most of them absorb light below 800 nm, limiting deep-tissue imaging applications. To this end, we utilized a recently described novel croconaine derivative (CR880) to develop CR880-based nanoparticles (CR880-NPs) for effective *in vivo* delivery, deep tissue optoacoustic imaging and photothermal therapy applications. Radicals and strong *π*–*π* stacking in CR880 result in an 880 nm absorption peak with no blue-shift upon condensing to the solid phase. DSPE-PEG2000-formulated CR880-NPs exhibited high optoacoustic generation efficiency and photostability, and could be visualized in the tumors of three different mouse tumor models (breast, brain, and colon tumor) with high image contrast. The high photothermal conversion efficiency of CR880-NPs (∼58%) subsequently enabled efficient *in vivo* tumor elimination using a low energy laser, while remaining biocompatible and well-tolerated. This work introduces a promising novel agent for cancer theranostics of challenging deep-seated tumors.

## 1 Introduction

Near infrared (NIR) light-triggered chromophores are a promising modality for photothermal therapy (PTT) due to favorable characteristics such as non-invasiveness, spatiotemporal control, negligible drug resistance, and low systemic toxicity [1–3]. Light-activated photothermal agents produce a thermal effect through nonradiative transitions [4–6], with the generated thermoelastic expansion from the tumor area subsequently producing ultrasonic signals which can be detected by optoacoustic imaging [7–9]. Therefore, photothermal agents which have both excellent optoacoustic and photothermal properties in the NIR region are highly desirable for *in vivo* cancer theranostics.

Various NIR light-absorbing organic agents and their nanoformulations have been recently developed for optoacoustic imaging-guided PTT, such as cyanine, squaraine, BODIPY, and tetrapyrrole dyes [5, 10]. However, these classes of dyes have inherent limitations such as multistep synthesis, low molar extinction coefficients, high quantum yield, and poor photostability, leading to weak optoacoustic signals and low heat generation [10, 11]. Croconaine dyes and their derivatives can overcome common limitations of other organic dyes considered for optoacoustic imaging and PTT [12]. For example, these agents exhibit a higher molar absorption coefficient, higher photostability, low quantum yield, with recent research further improving on their ease of synthesis, aqueous stability, and quenching [12, 13]. While several croconaine-based nanoparticles have been reported for *in vivo* optoacoustic imaging, most of them absorb light below 800 nm, limiting deep-tissue imaging applications [14–19]. Croconaine dyes absorbing longer wavelengths have also been reported [13], including a croconaine derivative absorbing in the NIR-II range (at 1052 nm) in the organic solvent DMF [20]. However, this compound is unstable in aqueous solvents, where it undergoes a blue-shift to 700 nm. Additionally, a croconaine derivative (A1094) with an NIR-II absorption maximum at 1094 nm was reported [21]. However, the achieved synthetic yield and the molar extinction coefficient were only 7.4% and 5.2*10^3^ in ethanol, respectively, with nanoformulation not improving the dye’s optical properties [22].

CR880 is a new member of the croconaine family recently reported for use in solar energy harvesting and water evaporation [23], and is able to absorb longer wavelengths. It has radicals and strong *π*–*π* stacking, and exhibits an 880 nm absorption peak with a strong molar absorption coefficient (1.84 × 10^5^ L mol^−1^ cm^−1^) and a 52% synthesis yield [23]. Importantly, CR880 does not undergo a blue-shift and quenching upon condensing to the solid phase. Therefore, proper nanoformulation of CR880 could take advantage of its excellent optical properties in an aqueous solvent, with great potential to be successfully utilized for deep *in vivo* applications.

To this end, we for the first time ultilized CR880 to develop CR880-based nanoparticles (CR880-NPs) for deep tissue optoacoustic imaging and PTT applications by loading hydrophobic CR880 into DSPE-PEG2000 to generate uniform nanoformulations. Similar to other croconaine-derived NPs we previously reported (CR760, CR780) [18, 19], CR880-NPs exhibit high optoacoustic generation efficiency (OGE), high photothermal conversion efficiency (PCE), and photostability as compared with various organic dyes or their derived nanoparticles, including the FDA-approved gold standard indocyanine green (ICG). Importantly, due to the longer absorption wavelength of CR880-NPs, they can be visualized from tissue deeper than any other croconaine dyes, with high image contrast and low background noise. We demonstrate that CR880-NPs can be passively and safely targeted to different mouse tumor models (subcutaneous breast cancer, orthotropic brain tumor, and orthotropic colon tumor), and can be visualized by multispectral optoacoustic tomography (MSOT) and subsequently used for MSOT-guided PTT using a low energy laser. We propose CR880-based nanoparticles as an efficient photothermal agent for deep tumor applications.

## 2 Results

### 2.1 Synthesis and characterization of CR880 and CR880-NPs

CR880 was prepared according to the reported method by condensation of two electron-donating segments with the electron-withdrawing croconic acid core to yield a “D-A-D” structure dye (Figure S1) [23]. The chemical structure of CR880 was confirmed by MALDI-TOF mass spectrometry and ^1^H NMR (Figures S2, S3). CR880 has moderate solubility in common organic solvents such as DMSO, tetrahydrofuran, chloroform, and ethFs1anol (Figure S4). CR880 shows a strong absorption peak at 880 nm with a significantly higher molar absorption coefficient in ethanol, which is consistent with other reported croconaine dyes [18, 19].

**Figure 1:**
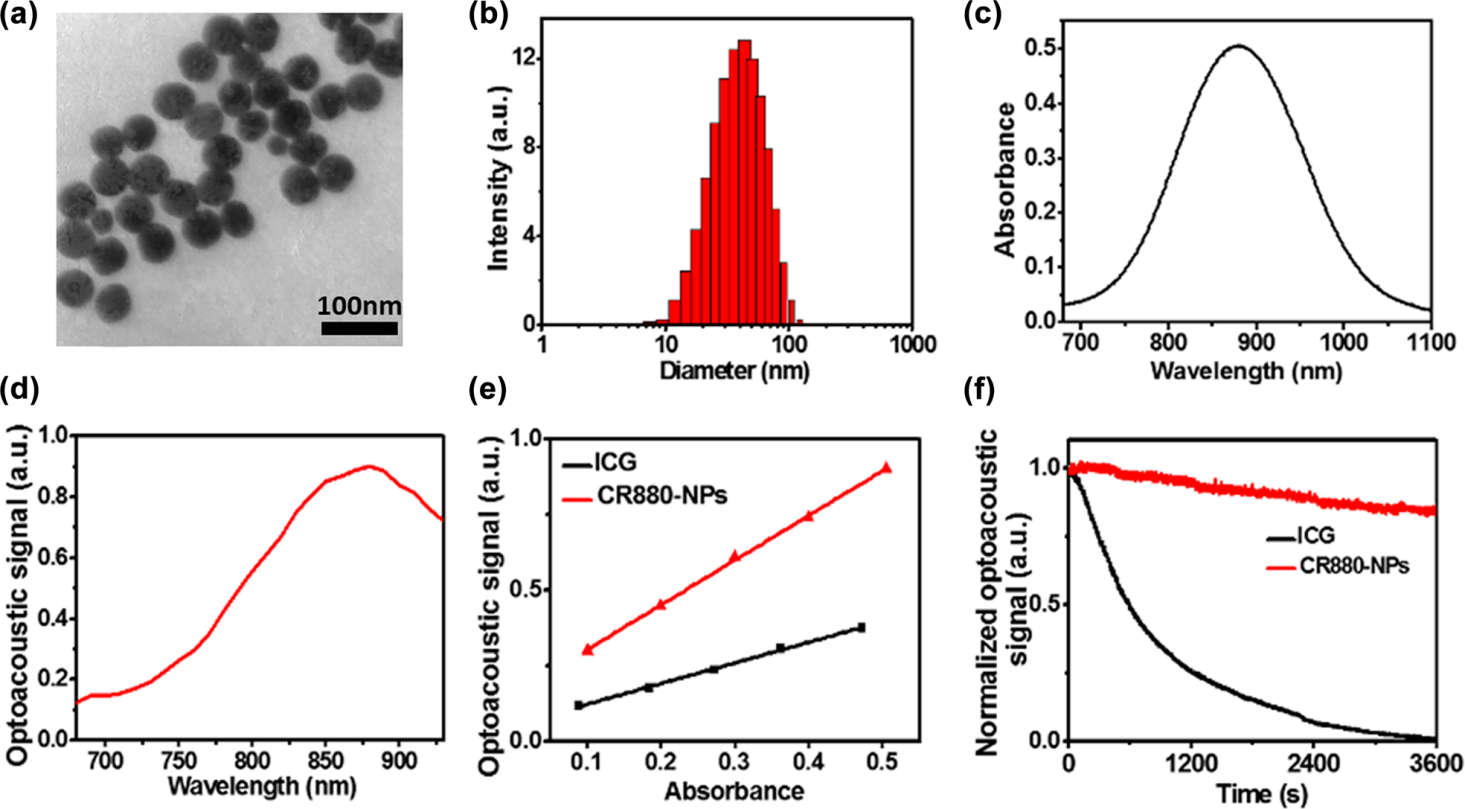
Physical characterization of CR880-NPs. The synthesized CR880-NPs as assessed by (a) transmission electron microscopy, (b) dynamic light scattering, (c) an optical spectrum obtained by spectrometry, and (d) an optoacoustic spectrum obtained by MSOT. (e) Optoacoustic signals of ICG and CR880-NPs at different concentrations, with the optoacoustic generation efficiency (OGE) indicated. (f) Optoacoustic signal degradation of ICG and CR880-NPs after pulsed laser irradiation (fluence ∼10 mJ/cm^2^, 60 min).

To apply hydrophobic CR880 for *in vivo* biomedical applications, we synthesized water-soluble CR880-NPs by the nanoprecipitation method with DSPE-PEG2000. The morphology and size of CR880-NPs were characterized by transmission electron microscopy (TEM) and dynamic light scattering (DLS). Figure 1(a) and (b) shows that the CR880-NPs have a spherical morphology with an average diameter of approximately 45 nm. Figure 1(c) and (d) shows the optical and optoacoustic spectrum of CR880-NPs with a narrow and intense peak at 880 nm. Next, Figure 1(e) demonstrates that the OGE of CR880-NPs was 2.16-fold higher than ICG, indicating that CR880-NPs can be an efficient optoacoustic agent. Furthermore, the photostability of CR880-NPs was assessed by continuous irradiation with a pulsed laser for 60 min, and the measured optoacoustic intensity, which can be used as an indicator of photostability, was compared against that of ICG. Figure 1(f) shows that CR880-NPs remained photostable while ICG was bleached entirely under the same irradiation conditions. Finally, a 14-day incubation in PBS and 10%FBS did not significantly alter the nanoparticle’s size and optical properties (Figure S5). These results suggested that the water-soluble CR880-NPs have excellent optical properties promising for *in vivo* optoacoustic imaging.

### 2.2 Optoacoustic imaging with CR880-NPs at different depths *in vitro*

**Figure 2:**
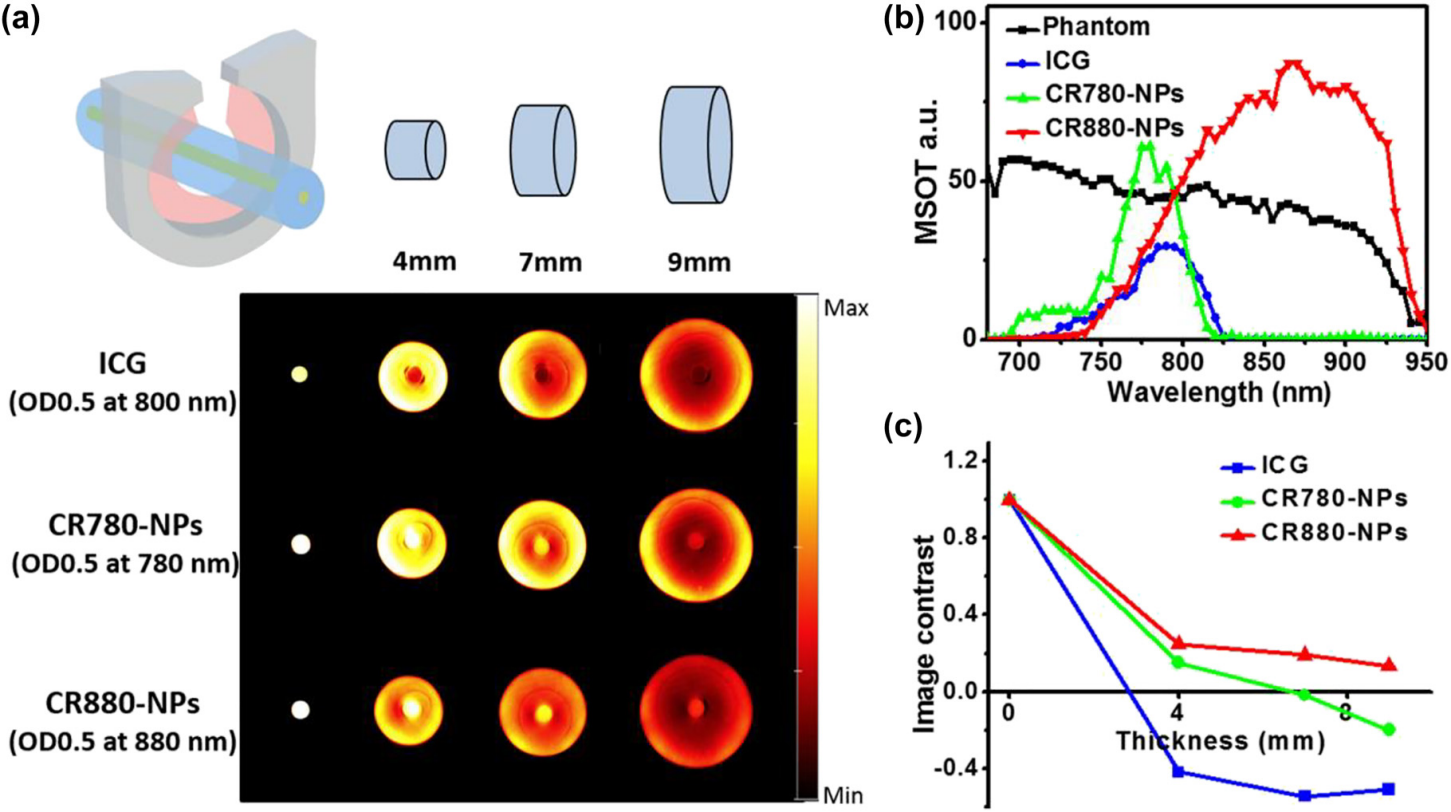
Optoacoustic imaging with CR880-NPs *in vitro*. (a) Optoacoustic imaging from the center of tissue-mimicking phantoms of increasing thickness containing different samples with the same absorption intensity (0.5) at their absorption peak. The optoacoustic images were individually acquired at their absorption peak as indicated. (b) Optoacoustic signals of ICG, CR780-NPs, and CR880-NPs in the 9 mm phantom. (c) Image contrast of ICG, CR780-NPs, and CR880-NPs in phantoms of different thickness.

To assess the capacity of CR880-NPs for deep tissue imaging, we evaluated the optoacoustic signals in tissue-mimicking phantoms of various thicknesses. Various cylindrical agar phantoms with different radii were prepared by mixing with India ink [24, 25]. A tube containing one of the samples (ICG, previously reported CR780-NPs [19], or CR880-NPs) at 0.5 absorbance was inserted into the phantom center, as illustrated in Figure 2(a). Agar phantoms containing CR880-NPs (880 nm absorption peak) displayed much lower noise signals compared to phantoms containing CR780-NPs (780 nm) and ICG (800 nm), while also exhibiting higher optoacoustic intensities at each thickness as exemplified in the reconstructed optoacoustic spectra in the 9 mm thick phantoms at their specific absorption peaks (Figure 2(b)). The concept of image contrast was used to reflect the imaging performance and depth, with CR880-NPs showing a much higher image contrast than the other two, even at the depth of 9 mm (Figure 2(c)). Furthermore, the optoacoustic signals of CR880-NPs were well correlated with nanoparticle concentration and phantom thickness, with the lower detection limit at 9 mm achieved at a 1.25 μM dye concentration (Figure S6). These results indicated the potential suitability of CR880-NPs for optoacoustic imaging of deep tissues.

### 2.3 *In vivo* optoacoustic imaging with CR880-NPs

**Figure 3:**
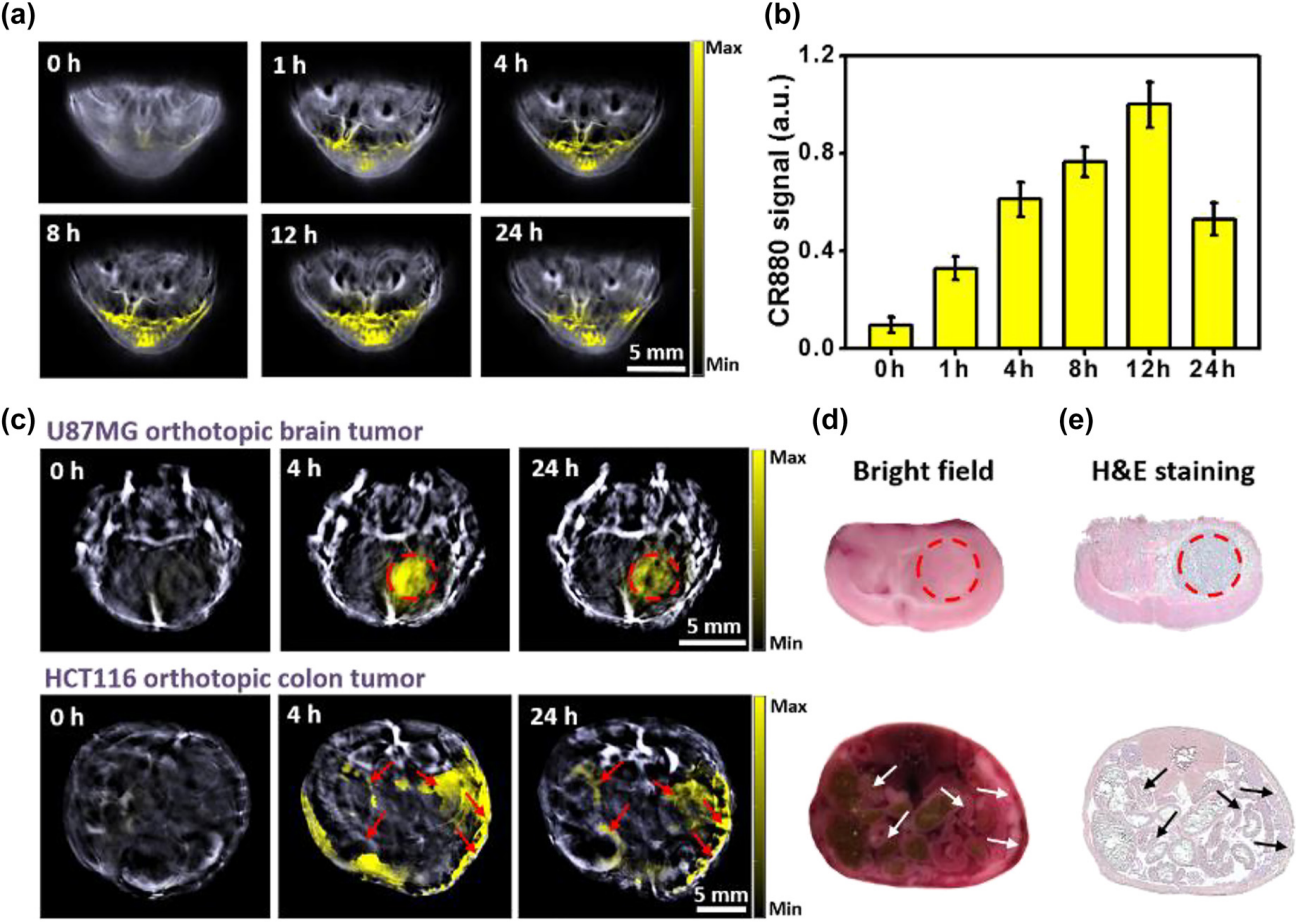
Optoacoustic imaging with CR880-NPs *in vivo*. (a) Representative unmixed MSOT images of 4T1 subcutaneous tumor models intravenously injected with 100 μL of CR880-NPs (0.3 mM), (*n* = 5). The unmixed CR880 signal is indicated by the yellow color in the tumor region. (b) Quantification of panel (a): CR880-NPs concentration in the tumor region measured over time. (c) Representative unmixed MSOT image of U87MG-bearing orthotopic glioblastoma and HCT116-bearing orthotopic colon tumor intravenously injected with 100 μL of CR880-NPs (0.3 mM) (*n* = 3). (d, e) Bright field imaging and H & E staining of a representative brain tumor (top) and colon tumor slices (bottom).

Building on the excellent optoacoustic image contrast in phantoms and the suitable nanoformulation, we further validated the capability of CR880-NPs for deep tissue imaging in three different mouse tumor models: a 4T1 subcutaneous tumor model and two orthotopic tumor models (brain and colon). Figure 3(a) shows representative unmixing optoacoustic images of 4T1 tumor-bearing mice, acquired using MSOT at different time points after nanoparticle injection (0 h, 1 h, 4 h, 8 h, 12 h, and 24 h). Enabled by their small nano-size and the enhanced permeability and retention (EPR) effect, accumulation of CR880-NPs could be observed in the tumor starting at 1 h post-injection, with the maximum accumulation seen at 12 h post-injection. However, optoacoustic measurement at 24 h post-injection revealed a decreased signal, indicating that CR880-NPs had undergone partial systemic clearance from the tumor region. Figure 3(b) shows that the signal intensities of the tumor region at 4 h and 12 h relative to 0 h were increased by a factor of 6.2 and 10.15, respectively, indicating that PTT is optimally conducted with a post-injection delay of 4–12 h.

Figure 3(c) shows representative optoacoustic images of orthotopic brain (U87MG) and colon (HCT116) tumors at 0 h and 24 h. MSOT images of brain tumors demonstrated that CR880-NPs can efficiently accumulate in the brain tumor. The optimal size distribution (20–70 nm) allows nanoparticles to penetrate through the disrupted blood–brain barrier (BBB) in the tumor region [19, 26], as the optoacoustic signal due to CR880-NPs was detectable from the brain tumor at depth of ∼5 mm. Additionally, MSOT imaging of the colon cancer models showed strong optoacoustic signals from the enterocoelia wall, where a curve shaped tumor tissue could be visualized. Moreover, the presence of CR880-NPs enabled the detection of colon tumors from a depth over 6 mm. The presence of the brain and colon tumor was validated by cryo-slicing and H & E staining (Figure 3(d) and (e)). These results provide evidence that CR880-NPs can generate efficient optoacoustic signals from deep tumors using the commercially available MSOT system.

At 24 h post-injection of CR880-NPs, the major organs (kidney, liver, spleen, and heart) and tumors from each tumor model were isolated for analysis of the *in vivo* biodistribution using MSOT imaging. Figure S7 shows the optoacoustic coronal plane images corresponding to signal intensities in major organs and tumors. These data indicated that most of CR880-NPs accumulated in the liver, spleen, and tumors, with a small degree of accumulation in the kidney and heart. Analysis of the blood clearance profile of CR880-NPs showed ∼8 h clearance half-life (Figure S8).

### 2.4 *In vitro* photothermal effect of CR880-NPs

**Figure 4:**
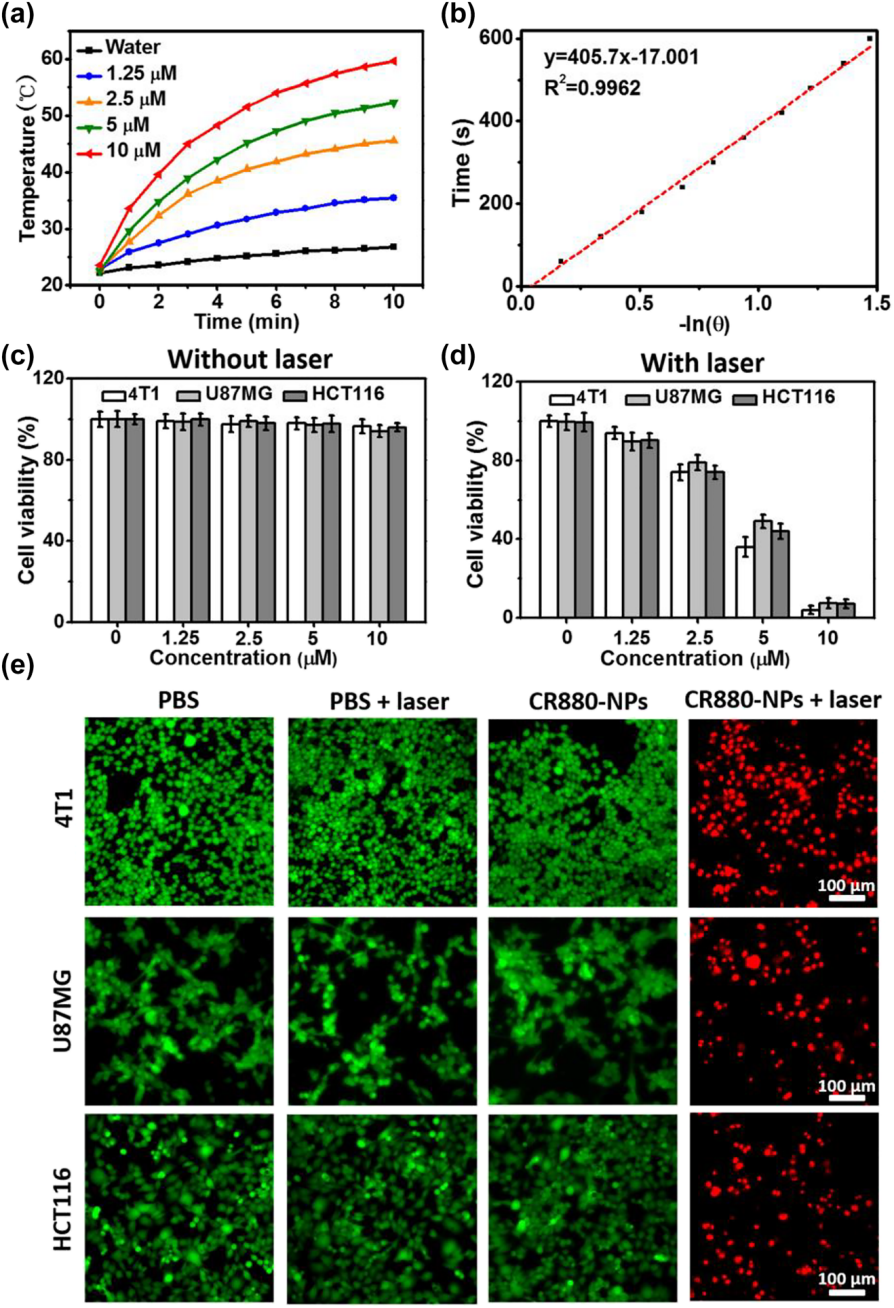
Photothermal effect of CR880-NPs *in vitro*. (a) Temperature change curves of CR880-NPs at different concentrations upon exposure to an 885 nm CW laser (0.8 W/cm^2^). (b) Linear correlation of the cooling times of CR880-NPs versus negative logarithm of temperature. (c, d) Relative viabilities of 4T1, U87MG, and HCT116 cells after treatment with CR880-NPs (10 μM) with and without 885 nm CW laser irradiation at 0.8 W/cm^2^ for 5 min. (e) Fluorescence images of the treated 4T1, U87MG, and HCT116 cells co-stained with calcein AM (green color, live cells) and EthD1 (red color, dead cells that remained adheared to the plates).

The strong NIR absorbance of CR880-NPs suggested that CR880-NPs could be an excellent photo-absorbing agent for PTT. To evaluate the PTT potential of CR880-NPs, we irradiated water and different concentrations of CR880-NPs (1.25, 2.5, 5, 10 μM) with an 885 nm continuous wave (CW) laser (0.8 W/cm^2^). Figure 4a shows that the temperature increase of irradiated CR880-NPs was concentration-dependent. Under the same laser irradiation, a 10 μM CR880-NPs solution underwent a 36.9 °C change, while water only increased by 5.2 °C. A 5 min treatment at 42–50 °C is known to kill cancer cells [27]. We therefore next explored the effect of laser power intensity (0.2, 0.4, 0.6, or 0.8 W/cm^2^) on the temperature change of CR880-NPs (10 μM). Figure S9 shows a clear laser power intensity dependence on the temperature increase. Figure 4(b) shows that CR880-NPs exhibited an average 58% photothermal conversion efficiency, which however depends on the concentration and molar absorptivity of the sample [27]. Additionally, CR880-NPs also showed excellent photothermal stability after 4 cycles of CW laser irradiation (Figure S10). No significant difference in photothermal stability was seen between pulsed laser and CW laser irradiated samples. To further evaluate the photothermal performance of CR880-NPs in deep tissue under 885 nm CW laser irradiation, different thicknesses of chicken breast tissue were placed on the CR880-NPs suspension and the temperature changes were monitored. CR880-NPs displayed a ∼12 °C, 9 °C and ∼6 °C temperature increase when a tissue thickness of 4 mm, 6 mm, and 8 mm was used, respectively, which is sufficient for treating deeply seated tumors (Figure S11).

To further verify the PTT potential of CR880-NPs in cancerous cell lines, we first tested the cellular uptake of CR880-NPs by measuring the optoacoustic signal of cell phantoms. Figure S12 show optoacoustic signal enhancements from 4T1, U87MG, and HCT116 cells treated with CR880-NPs for 4 h compared to untreated cells. Next, we used a standard MTT cell viability assay to assess the photothermal effect of CR880-NPs on these cells after incubation for 4 h. CR880-NPs showed negligible cytotoxicity at a concentration of up to 10 μM (Figure 4(c)). However, upon 5 min of irradiation with an 885 nm CW laser at 0.8 W/cm^2^, the viability of cells decreased in proportion to the concentration of CR880-NPs (Figure 4(d)). Specifically, more than 94% of cells were killed at a concentration of 10 μM under 0.8 W/cm^2^ laser irradiation. The PTT capability of CR880-NPs on the cells can also be visualized by live/dead cells assays. 4T1, U87MG, and HCT116 cells were subjected to different treatments for 4 h, and then co-stained with calcein-AM and EthD-1. Figure 4(e) demonstrates the significant difference in cellular cytotoxicity among the 4 treatment groups, in agreement with the MTT results above.

### 2.5 *In vivo* PTT of CR880-NPs in a 4T1 tumor model

**Figure 5:**
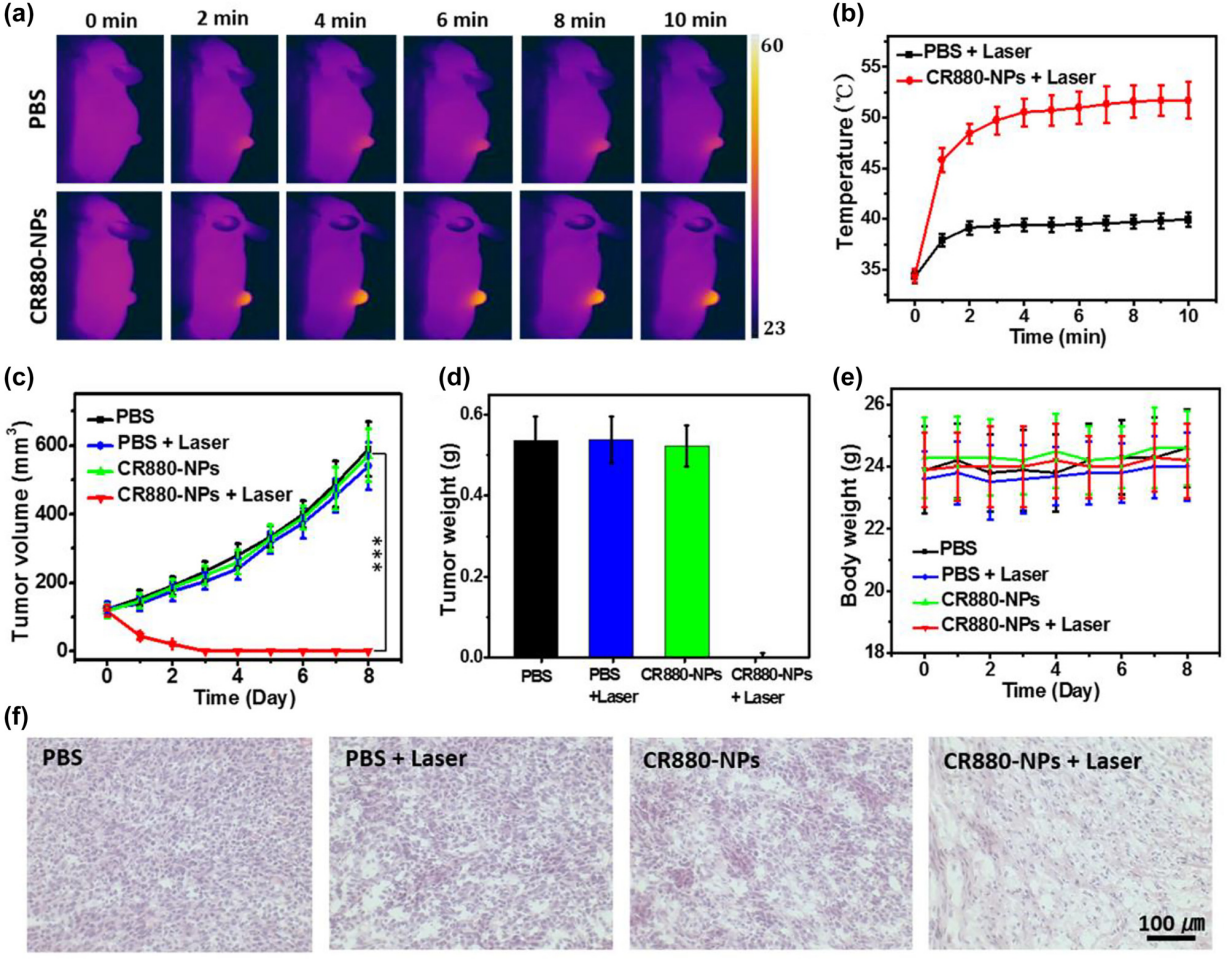
Photothermal effect of CR880-NPs *in vivo*. (a) Thermal images of 4T1 tumor-bearing mice after indicated treatments under laser irradiation (885 nm CW laser, 0.8 W/cm^2^). (b) The temperature changes of tumors recorded at different time points as shown in (a). (c–e) Relative tumor volumes, tumor weight, and body weight of mice having received different treatments. (f) H & E stained images of tumors resected from the different treatment groups at day 8. (***P < 0.001).

To evaluate *in vivo* tumor PTT using CR880-NPs, 4T1 tumor-bearing mice were randomly divided into four treatment groups: PBS, PBS + laser, CR880-NPs, and CR880-NPs + laser. We established 12 h as the optimum laser irradiation time, according to *in vivo* optoacoustic imaging of the 4T1 tumor model treated with CR880-NPs. The temperature changes of tumor areas were recorded by a thermal camera for 10 min. Figure 5(a) and (b) shows that the temperature of the tumor region injected with CR880-NPs reached 51.7 °C, which is sufficient to achieve cell elimination. However, mice treated with PBS showed negligible temperature changes under the same irradiation.

After treatment, the tumor volumes and body weights of all animals were continuously measured for another 8 days. As shown in Figure 5(c) and (d), the tumors from the group treated with CR880-NPs + laser displayed complete tumor elimination, while the tumor from the other 3 groups showed persistently high growth rates. These results suggested that CR880-NPs can be induced to generate significant heat for eliminating cancerous cells. The animal body weights did not show significant changes during the 8 days in any of the groups (Figure 5(e)). After 8 days, the residual tumor tissues and major organs from each group were resected and inspected by pathological examination by H & E staining. The CR880-NPs + laser treated group showed cell shrinkage, separation, and fragmentised nuclei, which indicated a therapeutic effect of CR880-NPs (Figure 5(f)). Additionally, the isolated vital organs from these treated mice did not show any organ damage (Figure S13). Altogether these results suggested that C880-NPs can be an efficient PTT agent for cancer treatment.

### 2.6 *In vivo* biosafety of CR880-NPs

To assess any potential side effects of CR880-NPs on organ function, we intravenously injected 100 μL of CR880-NPs (0.3 mM) into healthy C57BL/6 mice and collected blood and organ samples 7 and 14 days later for analysis. There were no evident differences in hematology and biochemistry parameters between animals treated with CR880-NPs and PBS (Figures S14a and b). H & E staining of vital organs (Figure S14c) further confirmed no detectable damage or inflammatory lesions due to CR880-NPs. Our results collectively indicate that CR880-NPs are biocompatible and well-tolerated *in vivo*.

## 3 Discussion

Here we described the nanoparticle formulation of the recently introduced CR880 dye for highly efficient deep tumor optoacoustic imaging and PTT. The highly sterically hindered and electron-donating tetraphenylethylene core of CR880 imparts it with the unusual property of not blue shifting upon aggregation. The formulated CR880-NPs exhibited high OGE, good photostability, and high PCE (∼58%). The longer wavelength and high OGE of CR880-NPs enable excellent image contrast from deep tissue with minimal background. Based on the EPR effect, CR880-NPs can effectively accumulate at the tumor region and be easily detected by optoacoustic imaging. Additionally, the photothermal performance of CR880-NPs was demonstrated by high-efficient ablation of cancer cells and by complete tumor inhibition in live mice. Finally, CR880-NPs were demonstrated to be biocompatible and nontoxic in mice.

In general, organic dyes are not soluble in aqueous systems; which often limits their biomedical applications. Nanoformulation makes it possible to solubilize highly lipophilic organic dyes [28]. For example, we previously developed PEGylated liposomes incorporating ICG which enabled efficient accumulation at the tumor site [29, 30], as well as encapsulation of the iBu-TSBSH5 dye into QH2 nanoparticles using a flash nanoprecipitation method [31]. Based on our previous work, we used DSPE-PEG2000 as the nanocarrier for the hydrophobic CR880. The formulated nanoparticles have a mean size of 45 nm, and can easily accumulate at the tumor region based on the EPR effect.

Despite the benefits of using NIR light for tissue imaging, achieving a high signal-to-noise ratio and depth of tissue penetration remain challenging because of absorption by hemoglobin and skin, and residual scattering in this wavelength range. Therefore, longer-wavelength dyes with optimized absorbance profiles are attractive targets. Most croconaine dyes or derived nanoparticles have absorption peaks around or below 800 nm [14–19]. Due to a diradical electronic configuration and strong *π*–*π* stacking of the coplanar structure, CR880 displays an NIR absorbance in the desired range of 880 nm. The longer wavelength absorption of CR880-NPs enabled high image quality and signal-to-noise ratio, enabling the straightforward detection of different tumor models, including the challenging brain tumor model.

Optoacoustic imaging resolves tissue images based on intrinsic tissue absorbers of light, such as hemoglobin, water, and lipids [32]. Contrast enhancement can be achieved with agents that exhibit a high absorption cross-section, high photostability, low quantum yield, low toxicity, and preferential bio-distribution and clearance profiles [33–36]. Exploiting the advantageous photophysical properties of croconaine dyes, CR880-NPs exhibit an efficient ability to absorb light energy for conversion into ultrasonic waves, while their photostability ensures persistent optoacoustic signal for disease detection. Combined with the sensitive MSOT system (spatial resolution: approximately between 100 and 150 µm), optoacoustic imaging of deep tumors can be therefore easily achieved. Concurrently, when contrast agents are irradiated with a laser, the generated thermal energy can be used for PTT of tumors. CR880-NPs show a PCE of 58%, much higher than most other photothermal agents [5, 37, 38].

In summary, croconaine-derived CR880-NPs represent a novel class of optoacoustic and photothermal agents for cancer theranostics, and pave a new way towards designing NIR light absorbing agents.

## 4 Methods

### 4.1 Materials

All the chemical reagents were purchased from abcr GmbH (Germany) unless noted otherwise. DSPE-PEG2000 was obtained from Nanocs Inc. (USA). Indocyanine green (ICG) and MTT were purchased from Sigma-Aldrich (Germany). CR780-NPs with 780 nm absorption peak were obtained from our previous work [19]. Calcein-acetoxymethyl (AM) and ethidium homodimer-1 (EthD1) were bought from Thermo Fisher Scientific.

### 4.2 Synthesis of CR880

CR880 was synthesized according to the previously described two-step procedure (Figure S1) [23]. Briefly, *in situ* boronation of bis(4-bromophenyl)amine with bis(pinacolato)diboron was followed by Suzuki coupling with the appropriate vinyl bromide in a one-pot procedure yielding a secondary amine in high yield. The sterically hindered amine then underwent Buchwald Hartwig coupling under forcing conditions, yielding the 2-amino thiophene which was then coupled with croconic acid under standard conditions to produce the desired dye. 400 MHz ^1^H NMR and MALDI-TOF were used for further characterization of the resulting product and found to be in accordance with the literature [23]. MALDI: *m*/*z* = 1625.826 [M + H]^+^.

### 4.3 Synthesis of CR880-NPs

CR880 (1 mg) and DSPE-PEG2000 (2 mg) were first dissolved in tetrahydrofuran (1 mL). Then the mixed solution was quickly injected into 9 mL deionized water and a microtip probe sonicator (12 W) was employed to sonicate the solution for 2 min, followed by removing the remaining tetrahydrofuran with a rotary evaporator. The final CR880-NPs were filtered by a 200 nm filter and then concentrated with a centrifugal-filter (MWCO = 100 kDa) for further use.

### 4.4 Characterization

^1^H NMR spectrum was measured on a Bruker spectrometer at 400 MHz. Mass spectrometry was recorded with an MALDI UltrafleXtreme (Bruker) using dihydroxybenzoic acid as the matrix. The particle dimensions of CR880-NPs were assessed by transmission electron microscopy (TEM) using a JEM 100-CX (JEOL GmbH, Germany) and dynamic light scattering (DLS) (Malvern Zetasizer). Absorption spectra were recorded with a UV-1800 spectrometer (Shimadzu, Japan). Optoacoustic spectra of samples were measured using an MSOT inVision 256-TF (iThera Medical, Munich, Germany) and then normalized with India ink and Brilliant Black BN [39]. The optoacoustic generation efficiency (OGE) of samples was calculated by the slope of the normalized optoacoustic intensities and absorbance of samples [38]. The photostability of samples was monitored by an MSOT inVision 256-TF with pulsed laser irradiation for 60 min (fluence 10 mJ/cm^2^). For OGE and photostability estimation, all samples were prepared in 10% fetal bovine serum (FBS) considering the quenching effects of ICG in water.

### 4.5 *In vitro* penetration depth estimation

Tissue-mimicking cylindrical phantoms with different radii were produced by using the mixture of intralipid (2 mL), agar (2 g), India ink solution (98 mL, absorbance 0.15 at 780 nm) [24, 25]. Then tubing containing ICG (0.5 at 800 nm), CR780-NPs (0.5 at 800 nm), and CR880-NPs (0.5 at 880 nm) were separately inserted into the ink-agar phantoms with different thicknesses. Optoacoustic phantom images were measured using MSOT. The image contrast was calculated by (OA_sig_ − OA_bg_)/(OA_sig_ + OA_bg_), where OA_sig_ and OA_bg_ are the mean optoacoustic intensities of sample and agar phantom at their specific wavelengths [40].

### 4.6 *In vitro* and *in vivo* optoacoustic imaging of various tumor models

Uptake of CR880-NPs by cells (4T1, U87MG, and HCT116) was evaluated by co-incubating nanoparticles for 4 h with cells cultured in a six-well plate. After 4 h, cells were washed thoroughly with PBS, scrapped and collected in an eppendorf tube. Next, alginate beads of cells were prepared following the previously described protocol for cell-alginate bead preparation for optoacoustic imaging [41]. Phantoms of CR880-NPs treatment and no treatment cells (control) were analyzed by optoacoustic imaging [41]. For *in vivo* optoacoustic imaging, 4T1 tumor models were prepared by implanting 4T1 cells (1 × 10^6^, 30 μL) on the back of 6-week old nude mice (*n* = 5). Upon reaching a tumor volume of 100 mm^3^, *in vivo* optoacoustic imaging was carried out. The orthotopic tumor models were generated using three 6-week old nod scid shorn mice by slowly implanting U87MG cells (4 × 10^5^, 3 μL) into the mouse striatum (bregma + 1.0 mm, left lateral 2.0 mm and depth 3.0 mm). The brain tumor models were ready after four weeks’ implantation [19]. The orthotopic colon tumor model was prepared with three normal 6-week old nude mice. The tumor model was ready for MSOT imaging two weeks after intraperitoneal injection of HCT116 cells (3 × 10^6^, 200 μL). All procedures involving animal experiments were approved by the Animal Care and Handling Office of Helmholtz Zentrum München and Government of Upper Bavaria.

Each tumor-bearing mouse per treatment was intravenouslyinjected with CR880-NPs (100 μL, 0.3 mM). *In vivo* optoacoustic images were taken at different time points before and after injection using an MSOT inVision 256-TF. The averaged optoacoustic signals of tumor regions were analyzed using ViewMSOT 4.0 software (iThera Medical, Munich).

### 4.7 *In vitro* photothermal effect of CR880-NPs

The photothermal conversion abilities of CR880-NPs were assessed by recording their temperature changes at different concentrations of CR880-NPs upon exposure to an 885 nm CW laser at different laser power using an IR thermal camera. The photothermal conversion efficiency (PCE) of CR880-NPs was calculated by the equation *η* = [(*hS*(*T* − *T*_surr_) − *Q*_Dis_]/*I*(1 − 10^−A885^). Here the absorbance (A885) of CR880-NPs was 0.18 at 885 nm and the power (I) of 885 nm CW laser was 0.8 W/cm^2^ [42].

4T1, U87MG, and HCT116 cells were sub-cultured in a 96-well plate overnight (1 × 10^4^ cells/well). The cells were first treated with PBS or different concentrations of CR880-NPs for 4 h, followed by washing with PBS and subsequent irradiation (885 nm CW laser) for 5 min, with the exception of the control. Then the cells were cultured for another 20 h to calculate the relative cell viabilities using the MTT assay. Live/dead cell assays were used to visualise the PTT effect at the cellular level. The treated cells were co-stained with Calcein-AM and EthD1 for 30 min and then imaged using the Leica DMI3000 B Inverted Microscope (Wetzlar, Germany).

### 4.8 *In vivo* PTT of 4T1 tumor-bearing mice

The 4T1 tumor-bearing mice with around 100 mm^3^ tumor volume were randomly divided into four groups (*n* = 5 per group) and subjected to 4 different treatments including: PBS, PBS + laser, CR880-NPs, and CR880-NPs + laser, separately (laser power: 0.8 W/cm^2^ 885 nm CW laser for 10 min, CR880-NPs concentration: 100 μL, 0.3 mM). An infrared thermal camera was used to record the temperature changes of tumor areas from each group. The tumor volume and body weight were measured for 8 days, after which the mice were sacrificed and the tumors and major organs from each group were isolated for H & E staining.

### 4.9 Blood hematology and biochemistry analyses

C57BL/6 mice were randomly divided into 3 groups (*n* = 5 per group). The control group was intravenously injected with 100 μL of PBS and blood collected on day 14. The other 2 groups were likewise injected with 100 μL of CR880-NPs (0.3 mM) and blood was collected on days 7 and 14. Hitachi 917 Clinical Chemistry Analyzer (Roche, Germany) was used to test the detailed parameters of blood hematology and biochemistry. The vital organs were isolated for H & E staining.

### 4.10 Statistical analysis

Statistical analysis was performed using OriginPro 8 (Northampton, Massachusetts, USA). Inter-group differences were assessed for significance using One-Way/Two-Way ANOVA with Tukey’s HSD test. Results were expressed as mean ± SD, and differences were considered significant if *P* < 0.05.

## Supplementary Material

Supplementary Material Details
