# Supplementary material for: Multifunctional croconaine nanoparticles for efficient optoacoustic imaging of deep tumors and photothermal therapy
Source: Nanophotonics. 2022 Sep 26;11(21):4637–47. doi: 10.1515/nanoph-2022-0469 (PMC11501461; doi:10.1515/nanoph-2022-0469)
Supplement: Supplementary file 1 — Supplementary Material Details [file j_nanoph-2022-0469_suppl_001.docx]

Supporting Information

**Multifunctional croconaine nanoparticles for efficient optoacoustic imaging of deep tumors and photothermal therapy**

Nian Liu,^1,2,3^ Patrick O'Connor,^4,5^ Vipul Gujrati,^1,2*^ Pia Anzenhofer,^2^ Uwe Klemm,^2^ Karin Kleigrewe,^6^ Michael Sattler,^5,7^ Oliver Plettenburg,^4,8^ Vasilis Ntziachristos^1,2,9*^

^1^ Chair of Biological Imaging, School of Medicine, Technical University of Munich, Munich 81675, Germany.

^2^ Institute of Biological and Medical Imaging, Helmholtz Zentrum München (GmbH), Neuherberg 85764, Germany.

^3^ PET Center, Department of Nuclear Medicine, the First Affiliated Hospital, Zhejiang University School of Medicine, Hangzhou 310003, China.

^4^ Institute of Medicinal Chemistry, Helmholtz Zentrum München (GmbH), Neuherberg 85764, Germany.

^5^ Institute of Structural Biology, Helmholtz Zentrum München (GmbH), Neuherberg 85764, Germany.

^6^ Bavarian Center for Biomolecular Mass Spectrometry (BayBioMS), Technical University of Munich, Freising 85354, Germany.

^7^ Bavarian NMR Center and Center for Integrated Protein Science Munich at Department of Chemistry, Technical University of Munich, Garching 85747, Germany.

^8^ Center for Biomolecular Drug Research (BMWZ), Institute of Organic Chemistry, Leibniz Universität Hannover, Hannover 30167, Germany.

^9^ Munich Institute of Robotics and Machine Intelligence (MIRMI), Technical University of Munich, Munich 80992, Germany.

^*^Correspondence: [vipul.gujrati@tum.de](mailto:vipul.gujrati@tum.de), [bioimaging.translatum@tum.de](mailto:bioimaging.translatum@tum.de)

**Figure S1.** Schematic of CR880 synthesis.


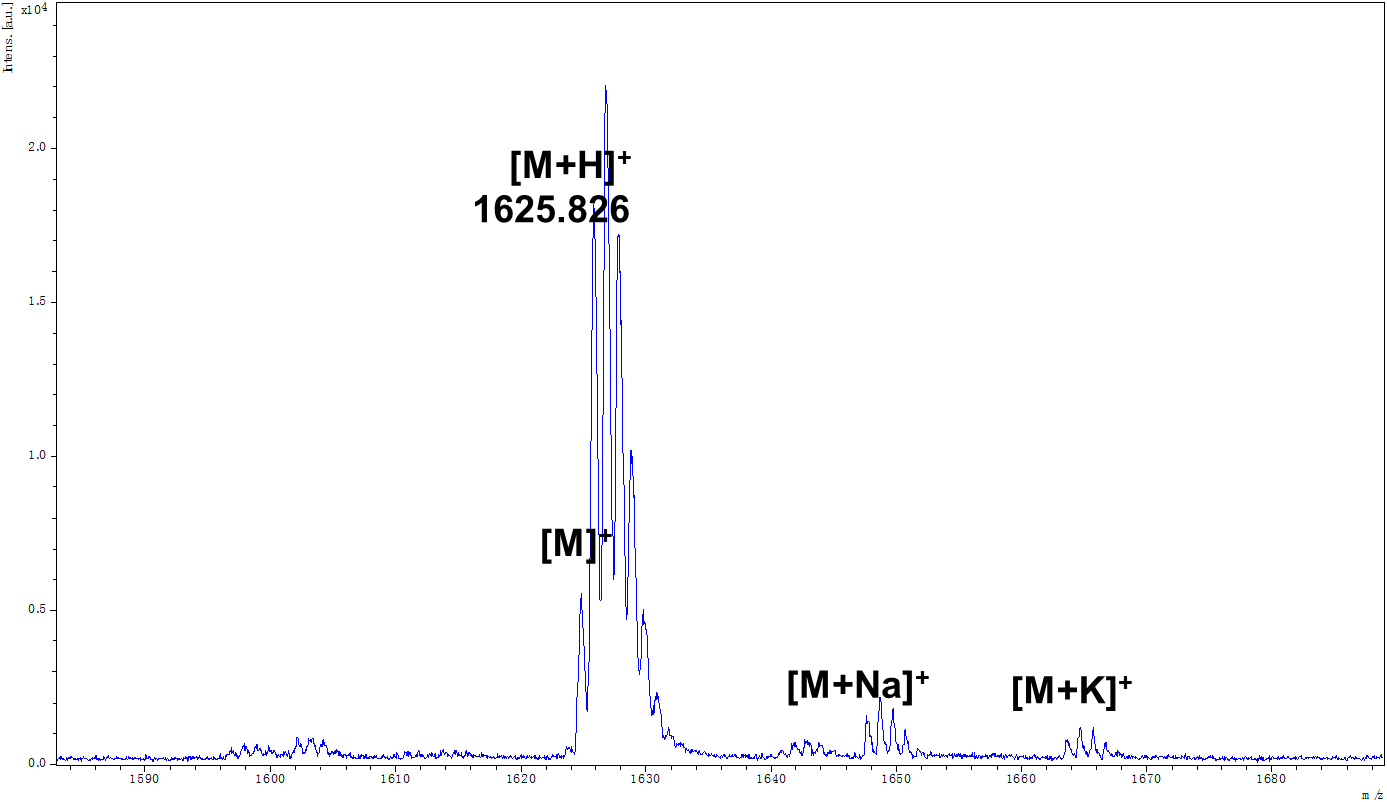


**Figure S2.** MALDI-TOF mass spectrum of CR880.


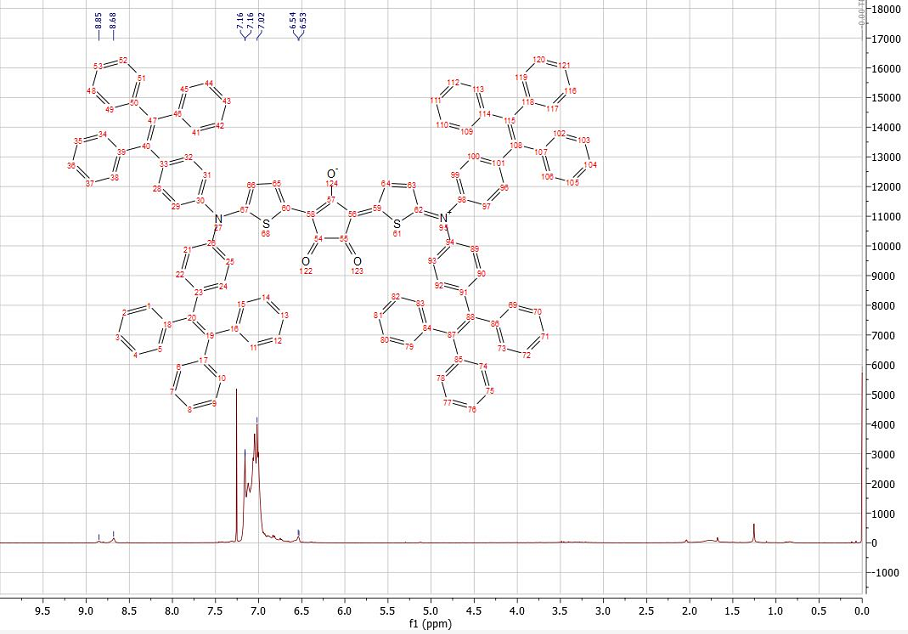


**Figure S3.** ^1^H NMR (400 MHz, chloroform-d_6_) of CR880.

**Figure S4.** Normalized optical spectra of CR880 in different organic solvents.


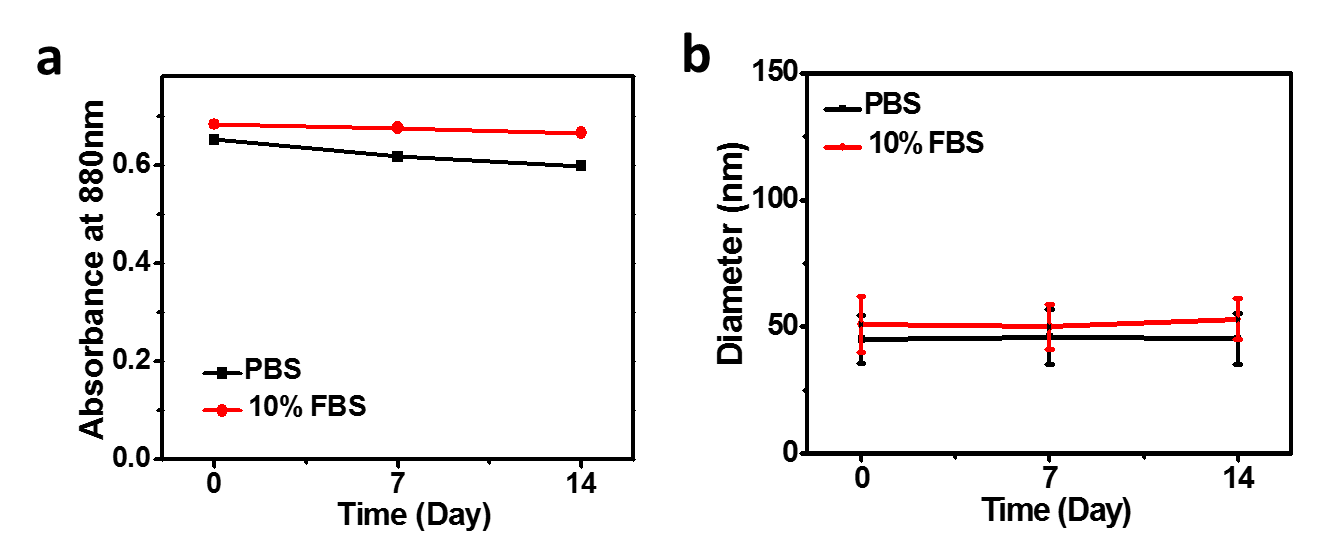


**Figure S5.** a) Optical stability and b) size stability of CR880-NPs in PBS and 10%FBS during 14 days.

**Figure S6.** Optoacoustic intensities of CR880-NPs at different concentration in phantoms of different thickness.


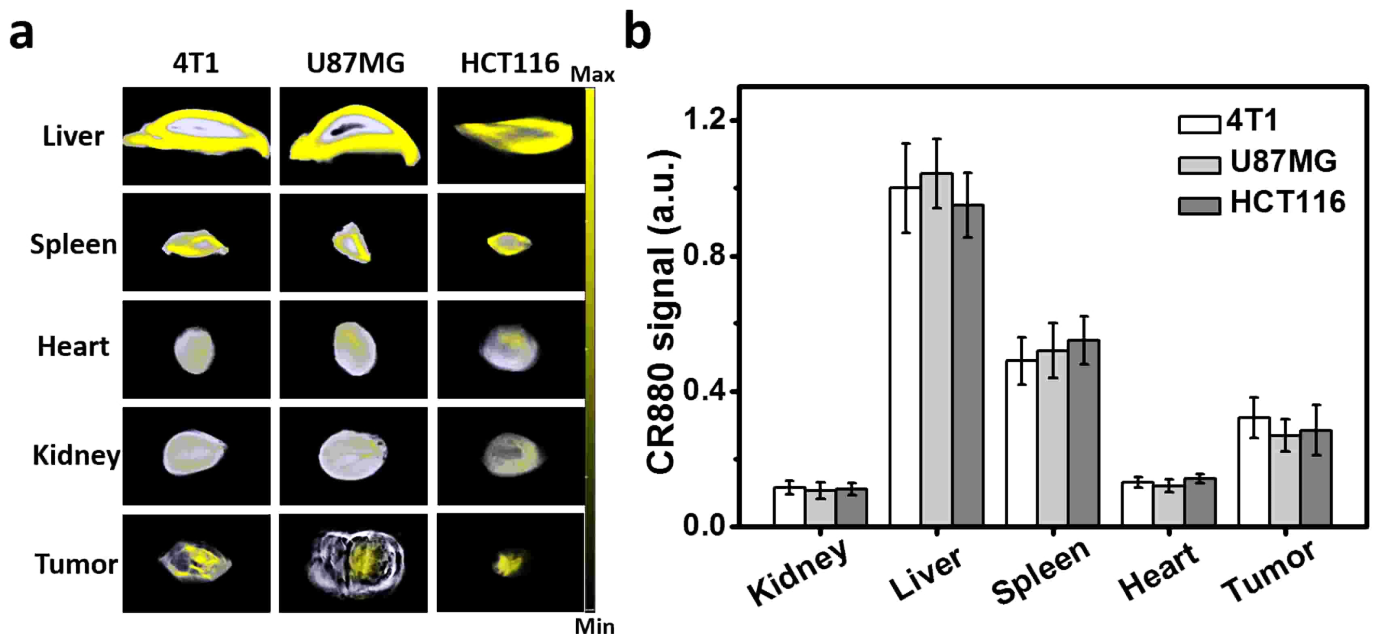


**Figure S7.** a) Optoacoustic coronal plane images of major organs 24 h after intravenous injection of CR880-NPs (0.3 mM, 100 μL) into different tumor models. b) Optoacoustic signal intensities of major organs.

**Figure S8.** Average concentration of CR880-NPs over time in mouse serum after intravenous injection of CR880-NPs (0.3 mM, 100 μL) and blood collection at various time points.

**Figure S9.** Temperature change curves of CR880-NPs (10 μM) upon exposure to an 885 nm CW laser at different power intensities.

**Figure S10.** Photothermal stability of CR880-NPs during four heating-cooling cycles (0.8 W/cm^2^ of CW laser; 20 min per cycle).

**Figure S11.** Temperature changes of CR880-NPs (10 μM) covered with various thickness of chicken breast tissues under CW laser irradiation (0.8 W/cm^2^).


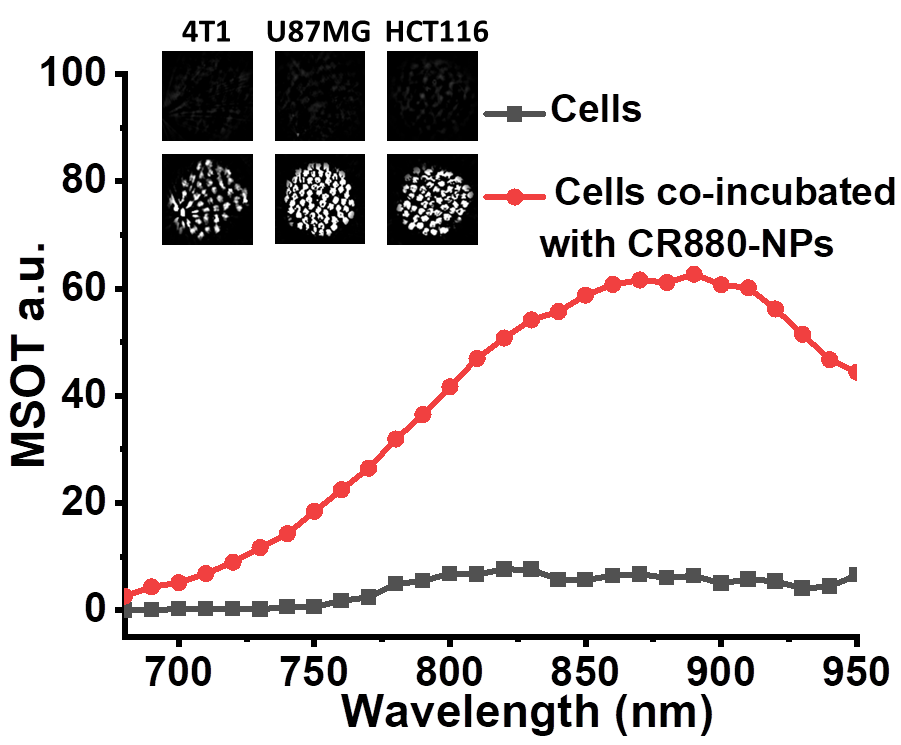


**Figure S12.** Optoacoustic images (at 880 nm) of cell-alginate beads phantoms without/with co-incubating CR880-NPs for 4 h and the corresponding optoacoustic spectrum.


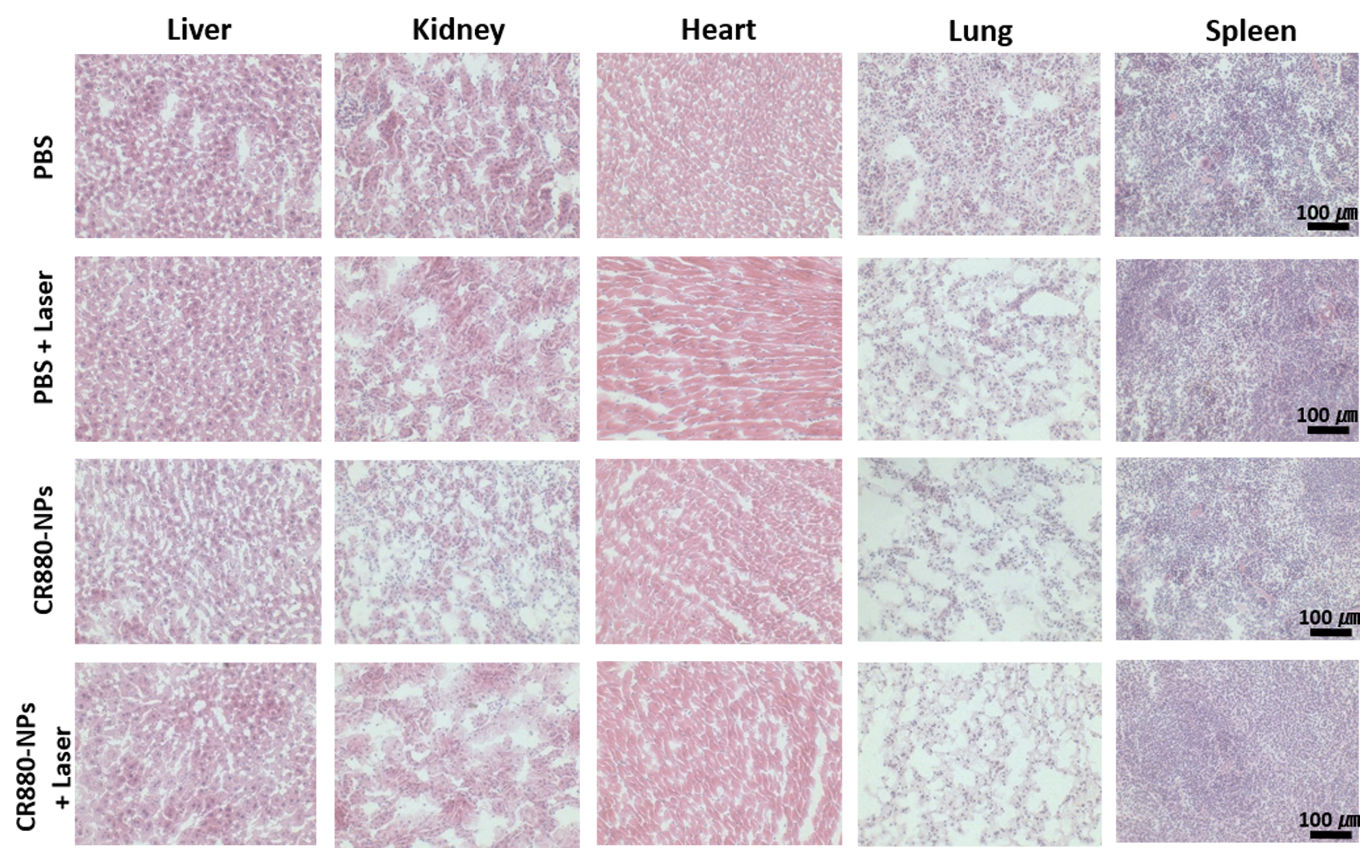


**Figure S13.** H&E staining of vital organs from nude mice after 8 days of treatment.


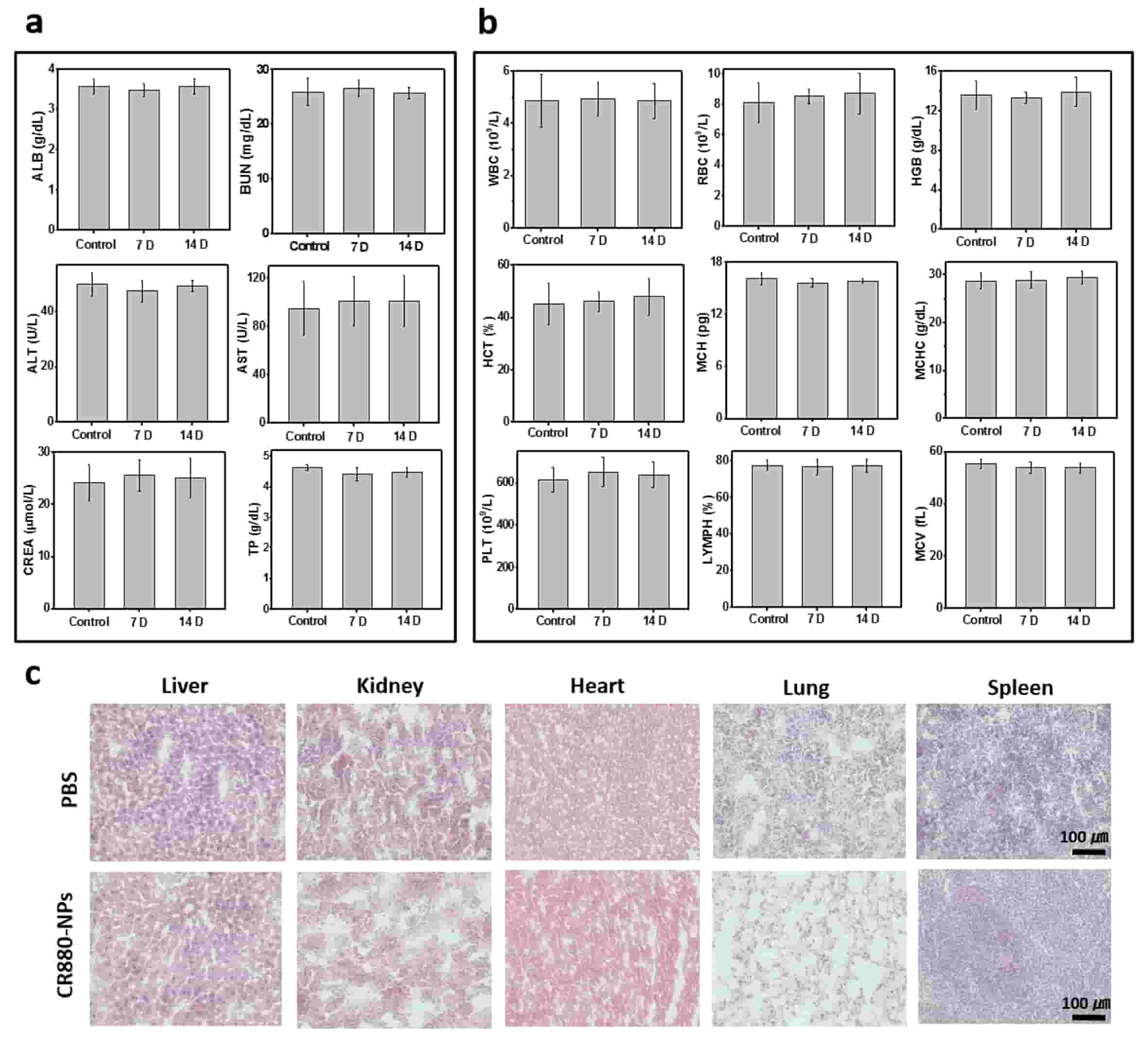


**Figure S14.** a) Blood biochemistry and b) hematology data of healthy C57BL/6 mice after intravenous injection of CR880-NPs (0.3 mM) or PBS (control) (n = 5 each). c) Representative H&E stained images of major organs collected from mice from the two groups 14 days after treatment. ALB, albumin; BUN, blood urea nitrogen; ALT, alanine transferase; AST, aspartate transferase; CREA, creatinine; TP, total protein; WBC, white blood cells; RBC, red blood cells; HGB, hemoglobin; HCT, hematocrit; MCH, mean corpuscular hemoglobin; MCHC, mean corpuscular hemoglobin concentration; PLT, platelet; LYMPH, lymphocytes; MCV, mean corpuscular volume.
